# The *Pelargonium sidoides* Extract EPs 7630 Drives the Innate Immune Defense by Activating Selected MAP Kinase Pathways in Human Monocytes

**DOI:** 10.1371/journal.pone.0138075

**Published:** 2015-09-25

**Authors:** Katrin Witte, Egon Koch, Hans-Dieter Volk, Kerstin Wolk, Robert Sabat

**Affiliations:** 1 Interdisciplinary Group of Molecular Immunopathology, Dermatology/Medical Immunology, University Hospital Charité, Berlin, Germany; 2 Berlin-Brandenburg Center for Regenerative Therapies, University Hospital Charité, Berlin, Germany; 3 Preclinical Research, Dr. Willmar Schwabe GmbH & Co. KG, Karlsruhe, Germany; 4 Institute of Medical Immunology, University Hospital Charité, Berlin, Germany; 5 Psoriasis Research and Treatment Center, University Hospital Charité, Berlin, Germany; 6 Research Center Immunosciences, University Hospital Charité, Berlin, Germany; Universitatsklinikum Freiburg, GERMANY

## Abstract

*Pelargonium sidoides* is a medical herb and respective extracts are used very frequently for the treatment of respiratory tract infections. However, the effects of *Pelargonium sidoides* and a special extract prepared from its roots (EPs 7630) on human immune cells are not fully understood. Here we demonstrate that EPs 7630 induced a rapid and dose-dependent production of TNF-α, IL-6, and IL-10 by human blood immune cells. This EPs 7630-induced cytokine profile was more pro-inflammatory in comparison with the profile induced by viral or bacterial infection-mimicking agents. The search for EPs 7630 target cells revealed that T-cells did not respond to EPs 7630 stimulation by production of TNF-α, IL-6, or IL-10. Furthermore, pretreatment of T-cells with EPs 7630 did not modulate their TNF-α, IL-6, and IL-10 secretion during subsequent activation. In contrast to lymphocytes, monocytes showed clear intracellular TNF-α staining after EPs 7630 treatment. Accordingly, EPs 7630 predominantly provoked activation of MAP kinases and inhibition of p38 strongly reduced the monocyte TNF-α production. The pretreatment of blood immune cells with EPs 7630 lowered their secretion of TNF-α and IL-10 and caused an IL-6 dominant response during second stimulation with viral or bacterial infection-mimicking agents. In summary, we demonstrate that EPs 7630 activates human monocytes, induces MAP kinase-dependent pro-inflammatory cytokines in these cells, and specifically modulates their production capacity of mediators known to lead to an increase of acute phase protein production in the liver, neutrophil generation in the bone marrow, and the generation of adaptive Th17 and Th22 cells.

## Introduction

Today, plant secondary substances, especially polyphenols, get more and more into the focus of medical research. In South Africa, polyphenol-rich herbal preparations made up from roots of *Pelargonium sidoides* and *Pelargonium reniforme* are traditionally used to treat respiratory and gastrointestinal infections, dysmenorrhea, and hepatic disorders [1]. Inspired by the healing of his tuberculosis, Charles Henry Stevens introduced this phytomedical drug to England already in 1897 [1]. More than seven decades later, a special ethanolic extract of *Pelargonium sidoides* roots, EPs 7630, was finally developed [EPs^®^ 7630 is the active ingredient of the herbal medicinal product Umckaloabo^®^ (ISO Arzneimittel, Ettlingen, Germany)]. In Germany, EPs 7630 is approved today for the therapeutic use in patients with acute bronchitis. In addition, EPs 7630 was shown to be effective in clinical trials with patients suffering from tonsillopharyngitis, rhinosinusitis, common cold or COPD [2–6]. As an alternative to antibiotic treatments, EPs 7630 has the advantage of not promoting microbial resistances [7]. The latter aspect is mainly explained by its characteristic not to interfere with the metabolism of viruses or bacteria.

The main constituents of EPs 7630 include coumarins (*e*.*g*., umckalin) and flavanoles (polyphenols) [8]. The latter comprise oligomeric proanthocyanidins, which are highly abundant (~40%) in EPs 7630, especially oligo- and polymeric prodelphinidins. They are constructed mainly of gallocatechin and epigallocatechin components and are present with different interflavonoid bonds in *Pelargonium sidoides* roots [9].

Although the research of the last years has made substantial progress with respect to the broad antiviral and antibacterial efficacy of EPs 7630, the exact mode of action of EPs 7630 is not fully understood yet. *In vitro*, EPs 7630 shows efficacy against cellular infections with influenza virus, HSV, EMCV, RSV, coronavirus, parainfluenza virus, and coxsackie virus, and this appears to be mainly mediated indirectly by inhibition of virus attachment and spreading [7, 10–13]. Different modes of action underlying the antibacterial effects of EPs 7630 have been proposed. EPs 7630 inhibits the adherence of bacteria such as *Streptococcus pyogenes* and *Helicobacter pylori* to epithelial cells *in vitro* [14–18]. Furthermore, ciliated cells isolated from the nasal epithelium enhanced their ciliary beat frequency in the presence of EPs 7630, which should allow a better removal of excess mucus and bacteria [19]. Regarding the infection with *Candida albicans*, EPs 7630 was shown to enhance the oxidative burst and intracellular pathogen killing by human blood phagocytes [15]. In *Leishmania major*-infected murine macrophages, EPs 7630 increased cellular nitric oxide production and mRNA levels of iNOS and several cytokines (IL-1β, IL-10, IL-12, IL-18, TNF-α, IFN-α, IFN-γ) [13, 20–22]. However, our knowledge regarding the influence of *Pelargonium* extract on human immune cells, in particular on their cytokine production, is still highly restricted. To gain insight into this matter we comprehensively studied the immunoregulatory effects of EPs 7630 on human blood immune cells.

## Materials and Methods

### Preparation of EPs 7630

EPs 7630 is prepared from the roots of *Pelargonium sidoides* with a drug to extract ratio of 1:8–10 using aqueous ethanol (11% w/w) as extraction solvent. Dried extract of a single batch (No. PSc2003/L01-11/SY06-041-A) was used to prepare a stock solution of 3 mg/ml. This solution was obtained by dissolving the powder in sterile PBS containing 10% ethanol, followed by ultrasound treatment and sterile filtration using a 0.2 μm filter unit.

### Cell Culture

Human peripheral blood mononuclear cells (PBMCs) were isolated from the blood of healthy donors by Ficoll (Biochrom) density gradient centrifugation as previously described [23, 24]. In the first setting, PBMCs were stimulated with EPs 7630 (3 and 30 μg/ml), *Escherichia coli* 0127:B8 lipopolysaccharide (TLR4 ligand; 100 ng/ml; Sigma-Aldrich), polyinosinic-polycytidylic acid [poly (I:C); 10 μg/ml; Sigma-Aldrich], a cytokine mixture of IL-1β, IL-2 and IL-12 (10 ng/ml each; R&D systems), anti-CD3 (Orthoclone; Cilag) and anti-CD28 (R&D systems) monoclonal antibodies (1 μg/ml each), or were left without specific treatment (0.1% ethanol as solvent control) for 4 and 24 h, before cell culture supernatant was recovered for ELISA cytokine production analysis. In another setting, PBMCs, after serum-starvation for 3.5 h, were stimulated with the same stimuli (for TCR stimulation CD3/28 coated Dynabeads were used at a cell / bead ratio of 2:1) but for 10 and 30 min, and cells were recovered for western blot analysis. For EPs 7630 dose-response analyses of cytokine production, isolated PBMCs were stimulated with EPs 7630 at concentrations ranging from 0.1 to 10 μg/ml or were left without stimulation (0.1% ethanol control) for 48 h. In some of these concentration-response analyses, lipopolysaccharide (LPS; 100 ng/ml) or poly (I:C) (10 μg/ml) was added after the first 24 h and incubation was continued for a further 24 h. To study the kinetics of cytokine production, isolated PBMCs were stimulated with 10 μg/ml EPs 7630 or 0.1% ethanol (solvent control) for 4 to 72h. For intracellular TNF-α analysis, PBMCs were stimulated for 4 to 5 h with EPs 7630 concentrations ranging from 1 to 30 μg/ml, with 0.1% ethanol solvent control and, if indicated, 25 ng/ml phorbol 12-myristate 13-acetate (PMA)/1 μg/ml ionomycin before being prepared for flow-cytometry-based cytokine analysis. In some of these studies, PBMCs were pretreated with SP600125, PD98059, wedelolactone (all from Sigma Aldrich) and SB202190 (Invivogen) at 10 μM each or with 0.1% DMSO (solvent control, Sigma Aldrich) for 45 min before EPs 7630 (10 and 30 μg/ml only) application. The possible influence of EPs 7630 on PBMC proliferation and apoptosis was tested by stimulation of PBMCs with 0 (0.1% ethanol solvent control), 3, 10 and 30 μg/ml EPs 7630 as well as with 1% DMSO (positive control for apoptosis induction) for 24 h. Afterwards, cells were recovered for flow cytometric analysis.

CD4^+^ memory T-cells were purified from isolated PBMCs by negative selection using the MACS system and the Memory CD4^+^ T-cell isolation kit (Miltenyi). For ELISA-based cytokine production analysis, isolated T-cells were cultured in the presence or absence of EPs 7630 for 48 h. For the last 24 h of culture, one part of these cells was stimulated with anti-CD3/anti-CD28-coated Dynabeads (Life technologies; cell/bead ratio 1:1).

All cell cultures were performed using RPMI culture medium supplemented with 2 mM L-Glutamin and 10% fetal bovine serum (Biochrom). The study of EPs 7630 effects, the collection and usage of the blood samples were approved by the clinical institutional review board of the University Hospital Charité Berlin, and written informed consent was obtained from donors.

### ELISA-based analyses of cytokine production

Culture supernatants were analyzed for TNF-α, IL-6 and IL-10 content by ELISA using respective detection kits from R&D systems. In some settings, quantification of TNF-α was carried out using the Immulite device and respective detection kit (DPC Biermann).

### Western blot analysis of signal transduction

Lysing of PBMCs, quantification of proteins in the cell lysates, SDS PAGE gel electrophoresis and blotting of the respective gel was carried out as published earlier [25, 26]. Analysis of signal transduction elements was performed by incubation of blots with antibodies detecting phospho-JNK1/2, phospho-p38, phospho-Erk1/2, phospho-Akt, phospho-STAT5 and phospho-p65 (all from Cell Signaling Technology) and GAPDH (Merck Millipore), followed by incubation with peroxidase-conjugated AffiniPure goat anti-rabbit or goat anti-mouse IgG (H&L, Dianova) and subsequent chemiluminescence detection using ECL reagent (Lumigen).

### Flow cytometry-based analyses

For analysis of intracellular cytokines, treated PBMCs were incubated with brefeldin A (Sigma Aldrich; 5 μg/ml) for the last 3 to 4 h of treatment. Afterwards, cells were fixed and permeabilized using the Cytofix/Cytoperm kit (BD Biosciences) and subsequently stained for 40 min with fluorescent antibodies detecting TNF-α (clone MAb11, BioLegend), CD14 (clone RMO52, Beckmann Coulter), and CD4 (clone SK3, BD Biosciences).

To assess the purity of isolated memory T-helper cells, cells were stained with fluorescent antibodies detecting the following cell surface markers as described previously [23, 24]: CD45RA (clone HI100), CD45RO (clone UCHL1), CD3 (clone SK7), CD4 (clone SK3), CD56 (clone NCAM16.2) (all from BD Biosciences) as well as CD16 (clone 3G8), CD14 (clone RMO52), and CD19 (clone J4.119) (all from Beckmann Coulter).

For analysis of PBMC proliferation, total cell numbers after treatment were assessed by flow cytometry.

For analysis of PBMC apoptosis, treated cells were stained with fluorescent annexin V antibodies and propidium iodide using the Annexin V apoptosis detection kit APC (eBioscience) according to the manufacturer’s instructions. Afterwards, the proportion of apoptotic cells (annexin V^+^ cells) were evaluated.

All data acquisitions and analyses were carried out using a FACSCalibur device and CellQuest software (BD Biosciences).

### Statistical Analyses

To test the significance of pairwise differences between the treatment groups, the Wilcoxon matched-pairs signed-rank test was used (SPSS software, IBM). A *p*-value of p ≤0.05 was considered as indicator of significance.

## Results

### EPs 7630 provokes rapid production of immune mediators

**Fig 1:**
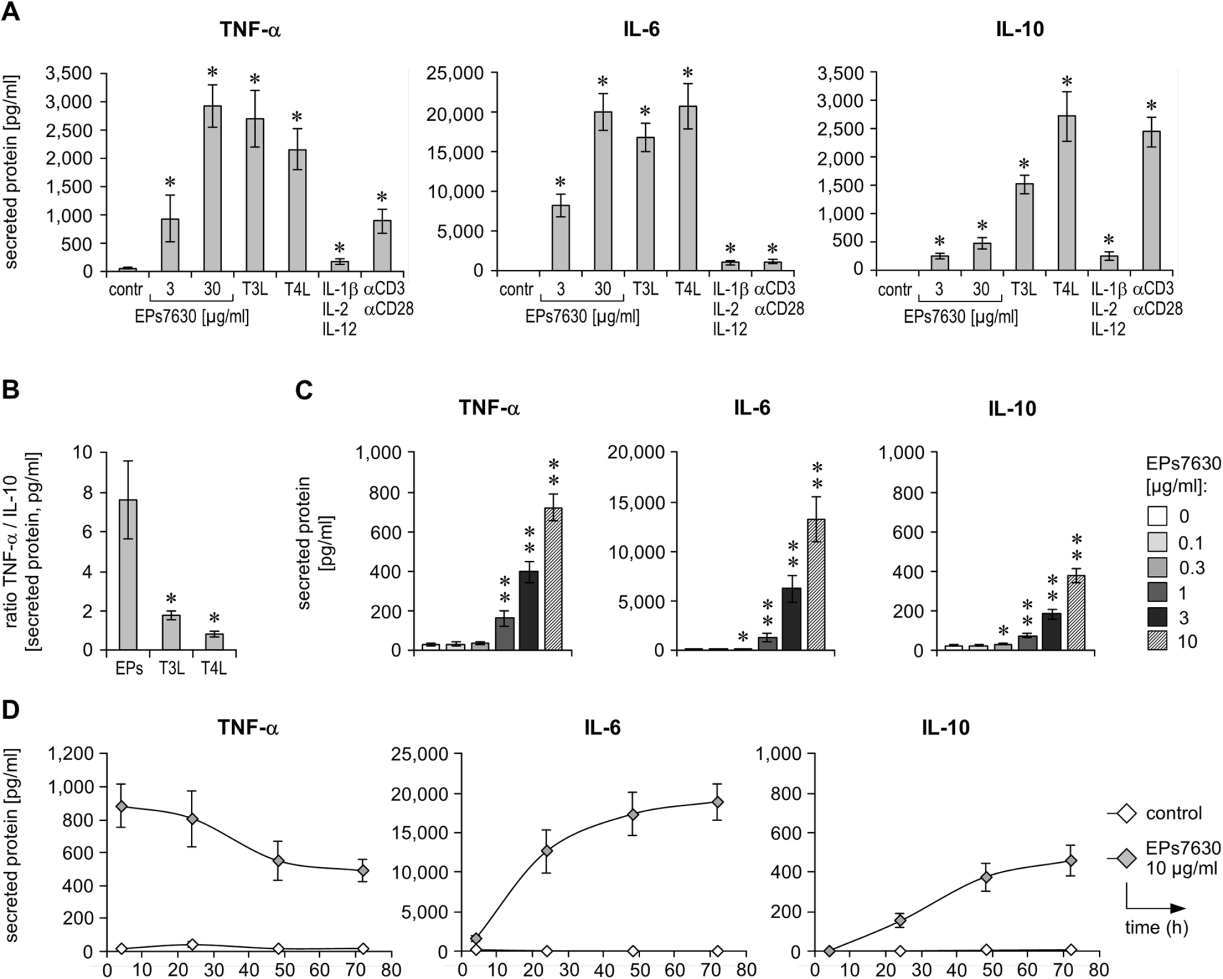
EPs 7630 stimulates human blood immune cells. (**A, B**) PBMCs, isolated from the blood of healthy donors, were treated with EPs 7630 as indicated, TLR3 and TLR4 ligands (T3L, T4L), a cytokine mixture (IL-1β, IL-2, IL-12), or anti-CD3 and anti-CD28 antibodies for 4 h and 24 h. TNF-α (4 h), IL-6 (24 h), and IL-10 (24 h) were quantified in respective culture supernatants by ELISA. Mean (± SEM) data from 6 donors are given as cytokine concentrations (A) or TNF-α/IL-10 ratio (B). (**C**) Healthy donor PBMCs were cultured in the presence of different concentrations of EPs 7630 as indicated for 48 h, followed by quantification of TNF-α, IL-6, and IL-10 in respective culture supernatants by ELISA. Mean (± SEM) cytokine concentration data from 12 donors are given. (**D**) Healthy donor PBMCs were stimulated with EPs 7630 and TLR4 ligand as indicated for 4 to 72 h, followed by quantification of TNF-α, IL-6, and IL-10 in respective culture supernatants by ELISA. Mean (± SEM) cytokine concentration data from 4 donors (except TNF-α 48h: n = 3) are given. (**A**, **B**) Significant differences compared to control group are indicated (* *p*<0.05, ** *p*<0.01, Wilcoxon matched-pairs signed-rank test).

Understanding the molecular effects of EPs 7630 is key to take advantage of its medical potential and the identification of further indications. So far, data regarding its target cells and effects thereof within the human immune system are lacking. To address this issue, we first investigated whether EPs 7630 influences resting human immune cells. Therefore, PBMCs isolated from healthy donors were treated or not (control) with EPs 7630 at two concentrations and were tested for cytokine production. As a comparison, these cells were also stimulated with toll-like receptor (TLR)3 [poly(I:C)] and TLR4 ligands (bacterial lipopolysaccharide), which mainly act on antigen-presenting cells (e.g., monocytes) and mimic viral and bacterial infection, respectively. As further positive controls a mix of cytokines (IL-1β, IL-2, IL-12), able to activate NK-cells, as well as T-cell activating anti-CD3/anti-CD28 antibodies, were used. Interestingly, cells responded to EPs 7630 by secretion of TNF-α and IL-6 with levels that were much higher than those induced by the mix of cytokines and TCR engagement (Fig 1A). Furthermore EPs 7630 slightly triggered IL-10 production, and this production was clearly smaller (~3- to 5-fold) than that induced by CD3/CD28-mediated stimulation. Importantly, the cytokine profile induced by EPs 7630 in PBMCs was also different from that induced by TLR3 or TLR4 ligands (Fig 1A). In fact, the EPs 7630-induced response was much more pro-inflammatory than that induced by the viral or bacterial infection-mimicking agents (Fig 1B). As no relevant cytotoxicity or influence on PBMC proliferation were observed by EPs 7630, the cytokine increase provoked by EPs 7630 seems to be due to an induced production rate (S1 Fig).

A more detailed investigation revealed a concentration-dependent induction of cellular TNF-α, IL-6, and IL-10 secretion by EPs 7630, with clear effects already seen at a concentration of 1 μg/ml (Fig 1C). Additionally, a kinetic analysis of EPs 7630-dependent cytokine production by PBMCs revealed, that especially TNF-α is produced very quickly (Fig 1D). Afterwards, the TNF-α level continuously decline, probably as a result of binding and internalization of this cytokine by monocytes. In contrast to TNF-α, IL-6 and, in particular, IL-10 were produced more slowly after EPs 7630 stimulation, with levels steadily increasing over time.

### T helper cells are not the main target of EPs 7630 action

**Fig 2:**
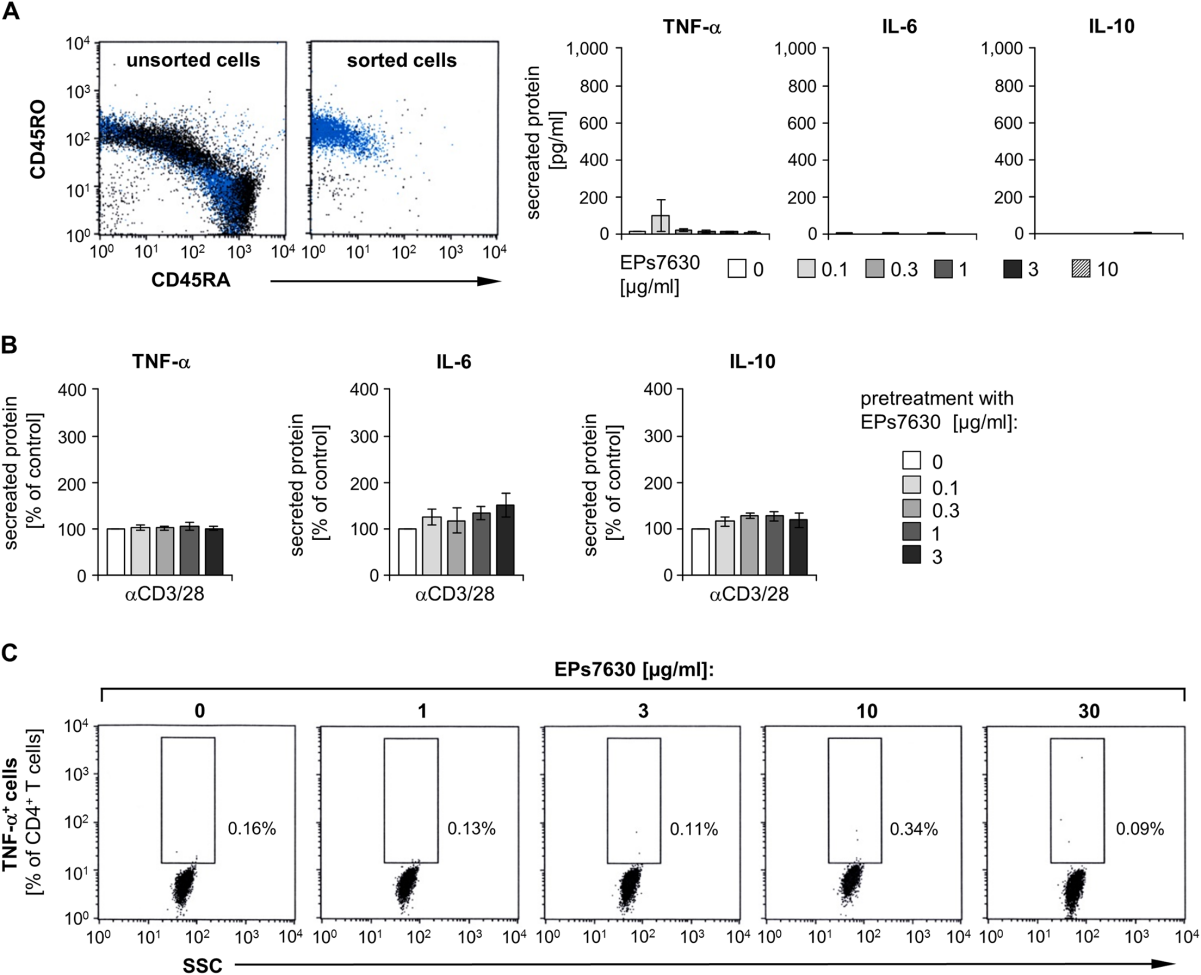
CD4^+^ memory T cells are not the main responders to EPs 7630. (**A**) Memory T-helper cells (CD4^+^ CD45RO^+^) were isolated from healthy donor PBMCs by magnetic labeling-based cell sorting. Representative dot plots of the cells (live gate cells with CD4^+^ lymphocytes marked in blue), stained and analyzed by flow-cytometry before and after sorting, are given (*left panel*). Mean (± SEM) purity of isolated cells was 97.68 ± 0.4%. Isolated cells were then treated with different concentrations of EPs 7630 as indicated for 48 h, followed by quantification of TNF-α, IL-6, and IL-10 in respective culture supernatants by ELISA. Mean (± SEM) cytokine concentration data from 5 donors are given (*right panel*). (**B**) Memory T-helper cells, isolated as in (A), were treated with different concentrations of EPs 7630 as indicated for 24 h. Subsequently, cells were additionally stimulated with anti-CD3/anti-CD28 antibodies for another 24 h. Quantification of TNF-α, IL-6 and IL-10 in respective culture supernatants was carried out by ELISA. Mean (± SEM) cytokine concentration data from 5 donors are given as percent of 0 μg/ml EPs 7630 group (control). Cytokine concentrations in the absence of EPs 7630 were: 4753±608 pg/ml (TNF-α), 3.4±0.9 pg/ml (IL-6), 737±103 pg/ml (IL-10). (**C**) Healthy donor PBMCs were stimulated with different concentrations of EPs 7630 as indicated for 4 to 5 h. Brefeldin A was added for the last 3 to 4 h of culture, followed by antibody-based staining of intracellular TNF-α and surface markers and subsequent flow-cytometric analysis. Representative dot plots showing the proportions of TNF-α^+^ cells out of all CD4^+^ cells of one out of 3 independent experiments are given.

The next study part aimed at identifying the cell type that accounts for the response observed in EPs 7630-stimulated blood immune cells. Since memory T helper cells are very important cytokine producers, we first isolated these cells from human PBMCs by magnet-based cell sorting (Fig 2A). Surprisingly, no relevant TNF-α, IL-6, or IL-10 production was found by these cells stimulated with different EPs 7630 concentrations (Fig 2A). In the next step, we tested whether EPs 7630 might modulate the cytokine response in activated memory T helper cells. However, CD3/CD28 stimulation of these cells, pretreated with different doses of EPs 7630, did not result in a relevant increase of TNF-α, IL-6, or IL-10 secretion (Fig 2B). Moreover, flow-cytometric analysis of intracellular cytokine staining showed that no population of T helper cells was able to produce TNF-α after stimulation with EPs 7630, whereas they strongly responded to PMA/ionomycin activation (Fig 2C and S2 Fig). These data suggest that, within PBMCs, not Th-cells but other immune cells are major targets of EPs 7630 action.

### EPs 7630 activates monocytes predominantly *via* selected MAPK pathways

**Fig 3:**
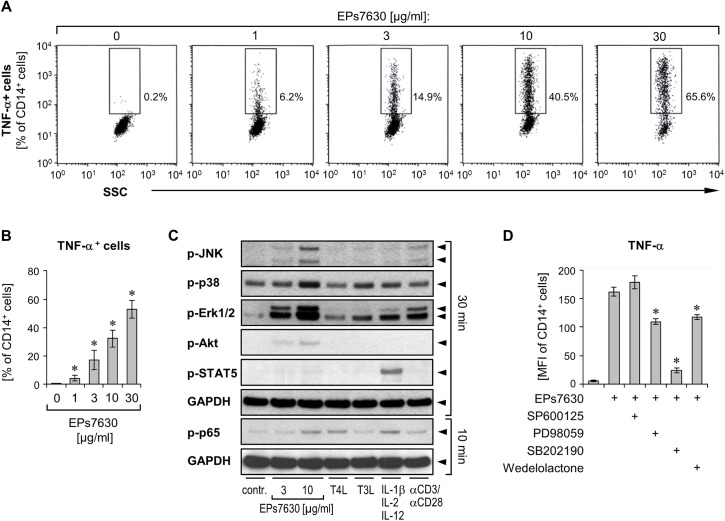
EPs 7630 mediates its effects via specific activation of MAPKs. (**A, B**) Healthy donor PBMCs were treated with different concentrations of EPs 7630 as indicated for 4 to 5 h. Brefeldin A was added for the last 3 to 4 h of culture, followed by antibody-based staining of surface markers and intracellular TNF-α and subsequent analysis by flow-cytometry. (A) Representative dot plots of CD14^+^ cells (monocytes) from one representative experiment are given, showing proportions of TNF-α^+^ cells out of all CD14^+^ cells. (B) Mean (± SEM) data of the proportions of TNF-α^+^ cells out of all CD14^+^ cells from 3 donors are given. Significant differences compared to control group (0 μg/ml EPs 7630) are indicated (* *p*<0.05, Wilcoxon matched-pairs signed-rank test). (**C**) Healthy donor PBMCs, starved for 3.5 h, were treated or not (control) with different concentrations of EPs 7630, TLR3 and TLR4 ligands (T3L, T4L), cytokine mixture (IL-1β, IL-2 and IL-12), and anti-CD3/anti-CD28 coated Dynabeads for 10 and 30 min as indicated. Activation of the phosphorylated signaling molecules JNK1/2, p38, Erk1/2, Akt, STAT5, and p65 as well as of the standardization marker GAPDH was assessed by Western blot analysis. (**D**) Healthy donor PBMCs were pretreated with inhibitors targeting MAP kinase and NF-κB signaling pathways [SP600125 (JNK1/2/3), PD98059 (MEK), SB202190 (p38α/β), wedelolactone (IκB kinase)] or a respective control solvent for 45 min, followed by addition of 10 μg/ml EPs 7630 and culture for further 4 h. Brefeldin A was added for the last 3 h of culture, followed by antibody-based staining of surface markers and intracellular TNF-α, and analysis by flow-cytometry. Mean fluorescence intensity (MFI) data of TNF-α signal in CD14^+^ cells (monocytes) are given from 5 donors as mean ±SEM. Significant differences compared to control group are indicated (* *p*<0.05, Wilcoxon matched-pairs signed-rank test).

To prove monocytes as being the cell population sensitive among PBMCs to the action of EPs 7630, we analyzed intracellularly stained TNF-α in these cells after EPs 7630 stimulation using flow cytometry. Indeed, we observed a strong dose-dependent induction of TNF-α production in monocytes, whereby, similar to the effect on whole PBMC, this effect was already seen at 1 μg/ml (Fig 3A and 3B).

To understand the action of EPs 7630 at the molecular level we then investigated the signaling pathways activated by this drug. PBMCs were treated for 10 and 30 min with EPs 7630 at two different concentrations (3 and 10 μg/ml) and, as comparison, with TLR3 and TLR4 ligands, the IL-1β, IL-2, and IL-12 comprising cytokine mixture, and anti-CD3/anti-CD28 antibodies. Because of the cytokine profile observed after PBMC incubation with EPs 7630, we focused our analysis on MAP kinase, NF-κB, and PI3K pathways. Respective Western blot analyses revealed that EPs 7630 activated the MAP kinases JNK1/2, p38, and ERK1/2. Additionally we observed a slight activation of the PI3K (phosphorylation of Akt) and NF-κB (phosphorylation of p65) pathways, whereas STAT5 expectedly remained unaffected (Fig 3C). In line with the different cytokine profiles induced by EPs 7630 versus TLR3 and TLR4 ligands, cytokine mixture, and anti-CD3/anti-CD28 antibodies, the pattern of activated kinases by EPs 7630 was unique. By pharmacological blocking using the selective inhibitors SP600125 (JNK1/2/3), PD98059 (MEK; activator of ERK1/2), SB202190 (p38α/β), and wedelolactone (IκB kinase), we observed that EPs 7630-induced production of TNF-α in monocytes was strongly dependent on p38 activity and at least partly dependent on the activation of Erk1/2 and NF-κB (Fig 3D).

### EPs 7630 modulates the immune response to bacterial and viral recognition

**Fig 4:**
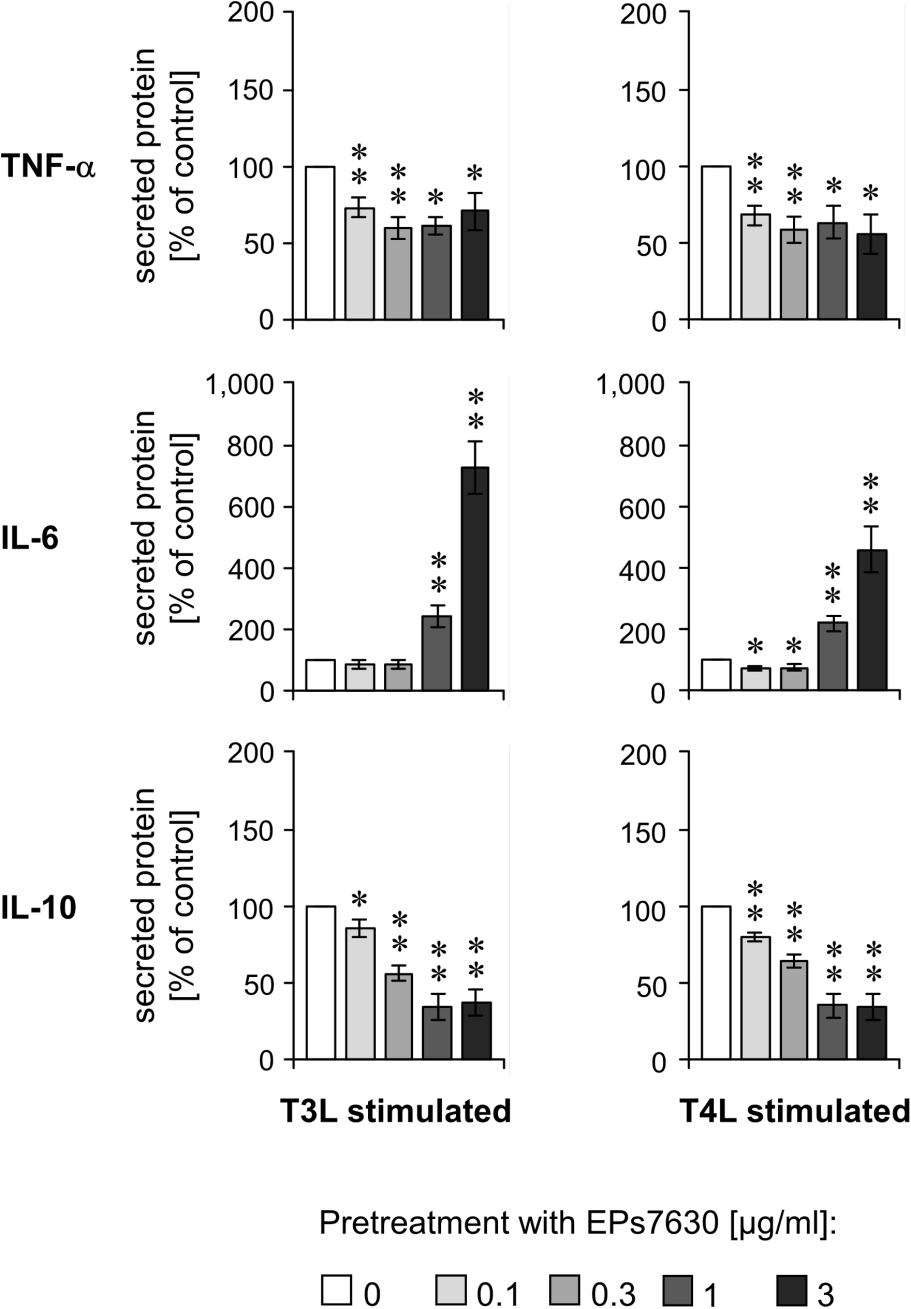
TLR3- and TLR4-induced responses in immune cells are modulated by EPs 7630 pretreatment. Healthy donor PBMCs were treated with different concentrations of EPs 7630 as indicated for 24 h. Afterwards, TLR3 or TLR4 ligands (T3L, T4L) were added for additional 24 h, followed by quantification of TNF-α, IL-6, and IL-10 in respective culture supernatants by ELISA. Mean (± SEM) cytokine concentration data from 12 donors are given as percent of 0 μg/ml EPs 7630 group (control). Cytokine concentrations in the absence of EPs 7630 in TL3L and TL4L groups were: 1267±414 and 1534±324 pg/ml (TNF-α), 1278±369 and 2135±650 pg/ml (IL-6), 1011±127 and 1430±140 pg/ml (IL-10), respectively. Significant differences compared to 0 μg/ml EPs 7630 group are indicated (* *p*<0.05, ** p<0.01, Wilcoxon matched-pairs signed-rank test).

EPs 7630 is very frequently used for infection prevention, in particular in the autumn and winter. Therefore, we asked whether EPs 7630 pretreatment would influence the immune response in the context of viral and bacterial infections. To address this question, we pretreated PBMCs with increasing concentrations of EPs 7630 (0.1–3 μg/ml) for 24 h followed by stimulation with TLR3 or TLR4 ligands (mimicking viral or bacterial infection, respectively) for another 24 h. Surprisingly, we observed that EPs 7630 pretreatment concentration-dependently inhibited the TLR3- and TLR4-mediated production of TNF-α and IL-10 (Fig 4). In contrast, we detected EPs 7630 concentration-dependent high IL-6 secretion in this situation (Fig 4). These data suggest that EPs 7630 pretreatment specifically modulates the cytokine production in subsequent viral and bacterial infection.

## Discussion

The special extract of *Pelargonium sidoides* roots, EPs 7630 (Umckaloabo^®^) is therapeutically active against upper respiratory tract infections and currently approved for the application in acute bronchitis in Germany. Surprisingly, the large use of this preparation is not associated with a respective knowledge regarding its pharmacodynamic properties. In fact, besides the known anti-infective properties of EPs 7630, directed against viruses and bacteria, little is known about its molecular effects and cellular targets. Moreover, we do not understand the pathway(s), *via* which EPs 7630 mediates its effects on cells. So far, the majority of published studies regarding the effects of *Pelargonium* extracts concern tissue cells. Only few studies involved immune cells, and most of them focused on murine macrophages [13, 15, 20–22]. Especially, there are no studies that investigated the influence of EPs 7630 on cytokine production by human primary immune cells. However, cytokines play an essential role in the defense against bacteria and viruses and in the intercellular communication between immune and tissue cells [27–32]. Therefore, the focus of our current study was to unravel the possible influence of EPs 7630 on human immune cells.

Our data show that EPs 7630 strongly and dose-dependently induced the production of the pro-inflammatory cytokines TNF-α and IL-6 in human blood immune cells. Moreover, a less prominent induction of the anti-inflammatory acting IL-10 was observed. Importantly, the cytokine profile induced by EPs 7630 was completely different from that induced by viral or bacterial infection-mimicking agents that caused production of a more anti-inflammatory cytokine milieu. These observations suggest that EPs 7630 acts as an immunostimulant and, before infection, may promote the innate immune defense and the ability of the body to quickly and efficiently eliminate potentially incoming microbes. Underlying mechanisms may include in particular the activation of phagocytes by TNF-α, the induction of acute phase proteins in the liver, and the elevation of neutrophil generation in the bone marrow.

Within human PBMCs, monocytes but not CD4^+^ T cells were found to be targets of EPs 7630 and to produce high amounts of TNF-α upon this treatment. This result is in line with previous data showing that *Pelargonium* extract acted on murine myeloid cells, in which it induced the production of TNF-α and IL-1β [13, 20–22].

When investigating the signaling cascades induced by EPs 7630, we found a strong MAPK kinase pathway activation, which included phosphorylation of JNK, Erk1/2, and p38. Furthermore, EPs 7630 slightly provoked NF-κB and PI3K pathway activation. However, pharmacological blockade of only p38 resulted in a strongly decreased monocyte TNF-α production. The observation that this signaling pattern differed from that induced by TLR3 and TLR4 ligand, inflammatory cytokines, and CD3/CD28 engagement suggest that *Pelargonium* extract affects monocytes *via* receptors different from those used by the mentioned stimuli.

It has previously been reported that the majority of *in vitro* macrophage activation with immune stimulating botanicals is caused by lipoproteins and lipopolysaccharide derived from bacterial contamination of the herbal raw material or from endophytes colonizing these plants [33]. As described above, we observed that the EPs 7630-activated pathway and cytokine pattern was very different from the response seen after stimulation of human PBMCs with bacterial lipopolysaccharide. Furthermore, analysis of EPs 7630 for the presence of contaminating bacterial lipopolysaccharide by a Limulus amebocyte lysate (LAL) assay revealed a very low content of less than 200 EU/mg, which is equivalent to about 20 ng/mg. Currently, it is not known which constituents of EPs 7630 are responsible for the observed effects on cytokine production. Previous investigations have suggested that the antiviral activity of the extract is primarily due to its content of prodelphinidins [7]. As these complex molecules may not be systemically bioavailable, further studies are required to demonstrate whether the clinically observed therapeutic effect of EPs 7630 is mediated by components different from prodelphinidins *via* stimulation of the immune system in the gastrointestinal tract.

By our study, we also demonstrated that EPs 7630 preincubation of immune cells influenced their subsequent, by viral or bacterial infection-mimicking agents induced cytokine production towards an IL-6-dominated milieu, with lower TNF-α and IL-10 content. This observation suggests that taking EPs 7630 before infection might reduce flu-like symptoms during infection, which are associated with the action of TNF-α. Furthermore, the EPs 7630 caused elevated IL-6 production during infection might strengthen the production of acute phase proteins, neutrophilic granulocyte generation in the bone marrow, and the establishment of adaptive Th17 and Th22 cells. In fact, the differentiation of Th17 cells is, besides IL-1β, IL-23 and TGF-β, particularly dependent on the presence of IL-6 [34]. Furthermore, IL-6 also cooperates with TNF-α in inducing the differentiation of Th22 cells [32, 35] and both Th17 and Th22 cells play an essential role in the defense against microbes at body barriers [36, 37]. Thus, by indirect induction of Th17 and Th22 responses, EPs 7630 treatment of patients may promote the adaptive host defense against different viral, bacterial or fungal pathogens at respiratory epithelia.

## Supporting Information

S1 Fig EPs 7630 does not influence apoptosis or proliferation of immune cells. **(A)** Healthy donor PBMCs were treated for 24 h with different concentrations of EPs 7630, 1% DMSO (positive control for apoptosis), or were left untreated (solvent control). Cells were stained with annexin V-specific antibodies and propidium iodide and subsequently analyzed by flow cytometry. Mean (± SEM) numbers of annexin V^+^/propidium iodide^-^ cells and annexin V^+^/propidium iodide^+^ cells are presented as percent of total PBMCs from 3 independent experiments. **(B)** Healthy donor PBMCs were treated for 24 h with different concentrations of EPs 7630 as indicated or were left untreated (solvent control). Cell counting was performed by flow cytometry. Mean (± SEM) data are given as percent of untreated cells as from 3 independent experiments.
(TIF)

S2 Fig PMA / ionomycin but not EPs 7630 provokes TNF-α production by memory T–helper cells. Healthy donor PBMCs were stimulated with EPs 7630, PMA / ionomycin or were left without stimulation (solvent control) for 4 h. Brefeldin A was added for the last 3h of culture. Afterwards, cells were subjected to antibody-based staining of intracellular TNF-α and cell surface markers and flow-cytometric analysis. Representative dot plots showing the proportions of TNF-α^+^ cells out of all CD4^+^ T-cells of one out of 2 independent experiments are given.
(TIF)
