# Supplementary figures and images for: The Pelargonium sidoides Extract EPs 7630 Drives the Innate Immune Defense by Activating Selected MAP Kinase Pathways in Human Monocytes
Source: PLoS One. 2015 Sep 25;10(9):e0138075. doi: 10.1371/journal.pone.0138075 (PMC4583277; doi:10.1371/journal.pone.0138075)

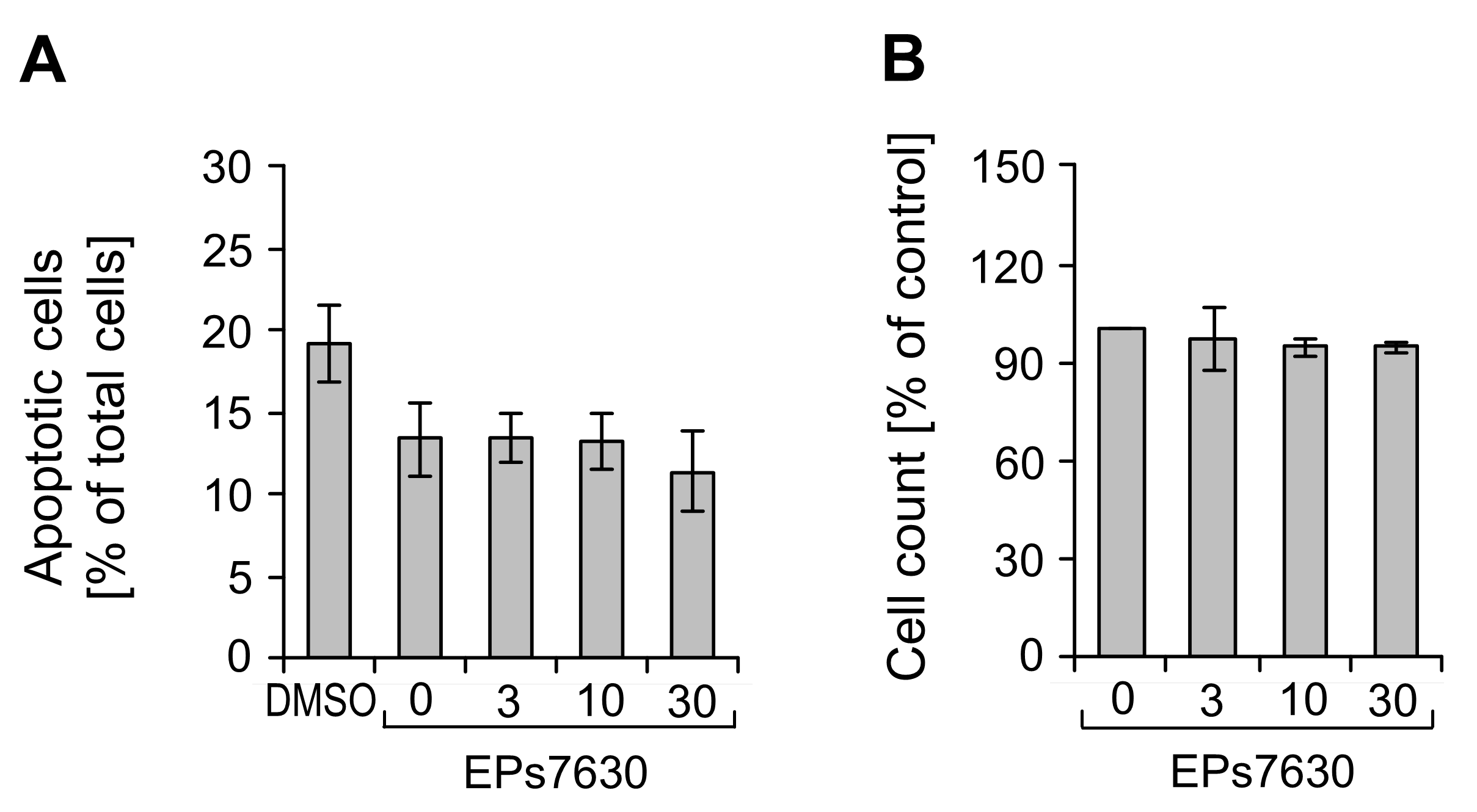

Supplement: S1 Fig — (A) Healthy donor PBMCs were treated for 24 h with different concentrations of EPs 7630, 1% DMSO (positive control for apoptosis), or were left untreated (solvent control). Cells were stained with annexin V-specific antibodies and propidium iodide and subsequently analyzed by flow cytometry. Mean (± SEM) numbers of annexin V+/propidium iodide- cells and annexin V+/propidium iodide+ cells are presented as percent of total PBMCs from 3 independent experiments. (B) Healthy donor PBMCs were treated for 24 h with different concentrations of EPs 7630 as indicated or were left untreated (solvent control). Cell counting was performed by flow cytometry. Mean (± SEM) data are given as percent of untreated cells as from 3 independent experiments. (TIF) [file pone.0138075.s001.tif]

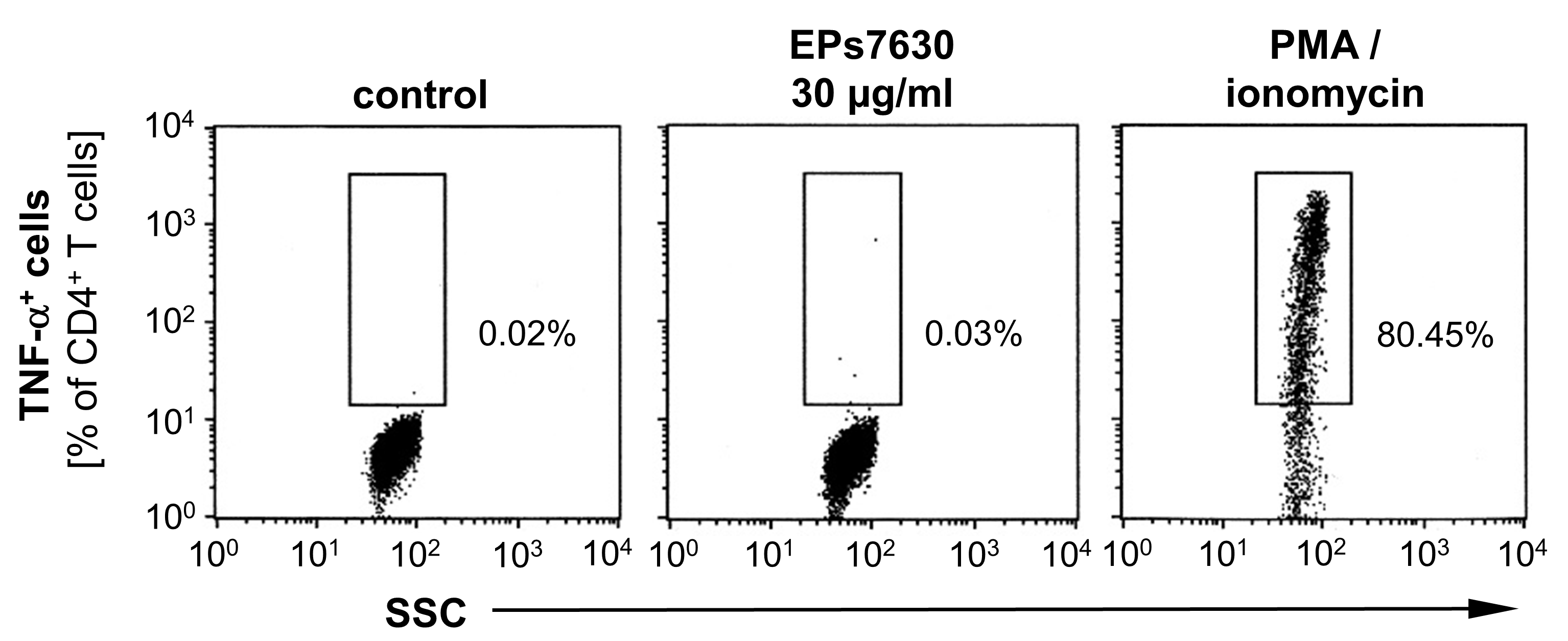

Supplement: S2 Fig — Healthy donor PBMCs were stimulated with EPs 7630, PMA / ionomycin or were left without stimulation (solvent control) for 4 h. Brefeldin A was added for the last 3h of culture. Afterwards, cells were subjected to antibody-based staining of intracellular TNF-α and cell surface markers and flow-cytometric analysis. Representative dot plots showing the proportions of TNF-α+ cells out of all CD4+ T-cells of one out of 2 independent experiments are given. (TIF) [file pone.0138075.s002.tif]
